# Parasitism modifies the direct effects of warming on a hemiparasite and its host

**DOI:** 10.1371/journal.pone.0224482

**Published:** 2019-10-30

**Authors:** Nicole E. Rafferty, Lindsey Agnew, Paul D. Nabity

**Affiliations:** 1 Department of Evolution, Ecology, and Organismal Biology, University of California, Riverside, California, United States of America; 2 Rocky Mountain Biological Laboratory, Crested Butte, Colorado, United States of America; 3 Department of Botany and Plant Sciences, University of California, Riverside, California, United States of America; University of Michigan, UNITED STATES

## Abstract

Climate change is affecting interactions among species, including host-parasite interactions. The effects of warming are of particular interest for interactions in which parasite and host physiology are intertwined, such as those between parasitic plants and their hosts. However, little is known about how warming will affect plant parasitic interactions, hindering our ability to predict how host and parasite species will respond to climate change. Here, we test how warming affects aboveground and belowground biomass of a hemiparasitic species (*Castilleja sulphurea*) and its host (*Bouteloua gracilis*), asking whether the effects of warming depend on the interaction between these species. We also measured how warming affected the number of haustorial connections between parasite and host. We grew each species alone and together under ambient and warmed conditions. Hosts produced more belowground biomass under warming. However, host biomass was reduced when plants were grown with a hemiparasite. Thus, parasitism negated the benefit of warming on belowground growth of the host. Host resource allocation to roots versus shoots also changed in response to both interaction with the parasite and warming, with hosts producing more root biomass relative to shoot biomass when grown with a parasite and when warmed. As expected, hemiparasite biomass was greater when grown with a host. Warmed parasites had lower root:shoot ratios but only when grown with a host. Under elevated temperatures, hemiparasite aboveground biomass was marginally greater, and plants produced significantly more haustoria. These findings indicate that warming can influence biomass production, both by modifying the interaction between host plants and hemiparasites and by affecting the growth of each species directly. To predict how species will be affected, it is important to understand not only the direct effects of warming but also the indirect effects that are mediated by species interactions. Ultimately, understanding how climate change will affect species interactions is key to understanding how it will affect individual species.

## Introduction

The elevated temperatures associated with global climate change are affecting species both directly and indirectly. Direct effects of warming include altered physiology, behavior, phenology, and distribution of species (e.g., [1,2]). Indirect effects can manifest as altered species interactions, via changes in the identities of interaction partners or the strength of interactions (e.g., [3,4]). Thus, to understand how climate change will affect individual species, it is important to determine both the direct and indirect effects of warming. Indeed, the indirect effects of warming via modified species interactions have the potential to outweigh the direct effects [5].

Understanding how warming will affect species involved in host-parasite interactions is of particular interest because the physiologies of host and parasite are often intertwined [6]. However, the effects of warming on hosts and parasites can be complex, complicating predictions of how climate change will alter their interactions [7,8]. If hosts accrue direct benefits from warming, such as faster growth, but in turn suffer indirect costs via increased parasitism, the net effect of warming may be negative or neutral (e.g., [9]). Conversely, hosts may perform more poorly under elevated temperatures, reducing certain fitness components, but be released from parasitism, increasing other fitness components. Similarly, the net effects of warming on parasites likely range from positive to negative and will depend on the host response [8].

Parasitic plants are important components of communities and larger ecosystems, acting as keystone species and ecosystem engineers in some contexts [10,11]. Hemiparasites are often generalists, interacting with a variety of host taxa by directly acquiring water, nutrients (including carbon and nitrogen), and secondary compounds from the xylem of host plants through haustorial connections, in addition to performing variable levels of photosynthesis [10–12]. Hemiparasites affect not only host performance [11,12] but also plant-herbivore and plant-pollinator interactions [13–16]. By changing competitive dynamics among species, hemiparasites influence plant community composition, and, at the ecosystem scale, alter decomposition and nutrient cycling [17,18].

Despite the important ecological role of hemiparasites, little is known about how climate warming will affect interactions with their host plants [8]. Under ambient air temperatures that ranged from 19–34 C over two months, carbohydrate levels in the root hemiparasite *Castilleja chromosa* did not vary with changes in microclimate, suggesting that the ability to extract sugars from hosts buffers the parasite from abiotic variation [19]. In another example, the facultative root hemiparasite *Euphrasia frigida* exhibited increased growth when temperatures were elevated by 2.3 C within open-top chambers, but whether this was due to direct effects of warming or indirect effects mediated by host plants was not determined [20]. If the direct effect of warming on parasitic plants is increased growth, they may compensate by taking more resources from their hosts, resulting in the interaction becoming more costly for the host under climate change [8].

Here, we focus on the interaction between *Castilleja sulphurea*, a hemiparasitic forb, and *Bouteloua gracilis*, a grass host. Because *C*. *sulphurea* is a facultative root hemiparasite, we can examine the effects of warming that are mediated by interactions with the host as well as the direct effects of warming on the parasite itself. Our overall aim was to test whether warming affects host and hemiparasite growth and to determine whether host-parasite interactions modify any direct effects of warming on the growth of individual species. We addressed the following questions: (1) How does warming affect growth of a hemiparasite and its host species? (2) Does the interaction between the hemiparasite and host modify the direct effects of warming on the growth of each species? (3) How does warming affect the intensity of parasitism as gauged by the number of haustorial connections between parasite and host?

## Materials and methods

### Study species

*Castilleja sulphurea* Rybd. (sulphur paintbrush; Orobanchaceae) is a perennial, facultative root hemiparasite that is native to the western United States and southwestern Canada. It grows in forests, by streams, and in subalpine meadows from 1950–3500 m, producing inflorescences with pale yellow bracts in the summer months [21]. The flowers are visited by bumble bees (*Bombus* spp.; [22]). Mature fruits (capsules) were collected from 20 haphazardly chosen plants at 2900–3100 m near the Rocky Mountain Biological Lab (RMBL) in Gothic, Colorado, USA in September 2016. All fruits were collected under special use permit (GUN1120) to RMBL from the USDA Forest Service, Gunnison National Forest.

*Bouteloua gracilis* (Kunth) Lag. (blue grama; Poaceae) is a perennial bunchgrass that flowers in the summer and is native to North America. It grows in a wide variety of habitats and at elevations of 1060–3050 m in Colorado, overlapping with *C*. *sulphurea* [21]. Bunchgrasses are known hosts of *Castilleja* species [23,24]. Seeds collected in 2017 from Montezuma County, Colorado, USA at 1828 m were obtained from a local seed supplier (Western Native Seed, Coaldale, Colorado, USA).

### Seed germination and early growth

All seeds were stored in a 4 C refrigerator until moist cold-stratification or direct seeding. *C*. *sulphurea* seeds were cold-stratified in spring 2018 to promote germination. Seeds were placed on moist filter paper in sealed Petri dishes in a 4 C refrigerator for ~120 days. Following stratification, seeds were sown in trays containing a standard soil mixture (57% sand, 43% peat moss, and various minerals) and grown in a growth chamber (Conviron MTR30) at 21 C, 50% relative humidity and 4.4 C, 20% relative humidity on a 12:12 h light:dark cycle. Seeds were monitored daily for germination, and germination date was recorded. In winter 2018, 66.0 ± 5.6 (mean ± SD) days post-germination, all live seedlings (174 plants) were selected for use in the experiment. At this stage, plants had 5.8 ± 2.6 pairs of leaves and were 12.8 ± 8.6 mm tall.

*B*. *gracilis* seeds were sown directly into pots containing the same soil mixture used for *C*. *sulphurea*. Three experimental growing conditions were established in 1 L pots: (1) one *C*. *sulphurea* seedling planted alone (“parasite alone”); (2) one *B*. *gracilis* seed planted alone (“host alone”); and (3) one *C*. *sulphurea* seedling and two *B*. *gracilis* seeds planted together, ~3 cm apart (“host + parasite”). The two *B*. *gracilis* seeds were monitored for germination to ensure that only one host was present; if both seeds germinated, the later germinant was removed from the pot.

### Temperature treatments

All experimental plants were assigned to one of two greenhouse bays programmed to achieve different temperatures at the University of California, Riverside. Temperature treatments were designed to approximate (1) ambient July air temperatures near the site of *C*. *sulphurea* seed collection (Rocky Mountain Biological Lab, Gothic, Colorado, USA) and (2) these summer air temperatures elevated by 3 C on average. Warming of 3 C represents the projected global temperature increase by 2100 under current mitigation goals [25]. The mean July air temperature in Gothic from 2016–2018 was 12.7 C [26]. Supplemental lights (Pro 1000^e^ 120/240 DE US, Gavita Holland bv) were programmed to turn on in each bay from 1630–2100 to extend the ambient photoperiod to 14 h of light to match the photoperiod of Gothic in July. Temperatures throughout the study were measured at 15 min intervals with data loggers (HOBO MX2302, Onset). Plants were watered as needed to maintain adequate soil moisture. In total, 20 “parasite alone” pots, 36 and 24 “host alone” pots, and 67 “host + parasite” pots were assigned to the ambient and warmed temperature treatment, respectively.

### Biomass measurements

Seventy days after being assigned a temperature treatment, plants were destructively harvested for biomass measurements. Plants were soaked in water to loosen and rinse away soil from the roots. For plants in the host + parasite growing condition, the number of haustorial connections (S1 Fig) was counted under a dissecting microscope before the host and hemiparasite root systems were separated. Aboveground and belowground wet biomass of each plant was separated, placed in a drying oven for 24–48 h at 50 C, and weighed to the nearest 0.1 mg.

### Statistical analyses

We used linear models to test whether the temperatures in our ambient and warmed greenhouse bays differed. We fit five models with different temperature metrics as the responses: all data, daytime, nighttime, daily maximum, and daily minimum. Temperature treatment (ambient vs. warmed) was the predictor.

To determine how growing condition (host/parasite alone vs. host + parasite) and temperature treatment (ambient vs. warmed) affected biomass, we used linear models. We fit separate models with host (or parasite) aboveground (or belowground) biomass as the response variable and growing condition, temperature, and their interaction as predictors. Using these same predictors, we also fit models with the ratio of host (or parasite) belowground to aboveground biomass (root:shoot) as the response variable. To achieve normality, aboveground and belowground biomass data were log-transformed, and we applied a logit transformation to the ratio of belowground to aboveground biomass. To assess multiple coefficients in the models simultaneously, we used likelihood ratio (LR) tests. We report only the best-fitting models.

To examine biomass allocation patterns for plants in the host + parasite growing condition, we regressed aboveground biomass against belowground biomass for individual host plants. We repeated this analysis for individual hemiparasites. We also regressed aboveground (or belowground) biomass of individual host plants against aboveground (or belowground) biomass of the individual hemiparasites with which they were grown.

We used a chi-square test with Yates continuity correction to test whether hemiparasites were more or less likely to develop haustoria in the ambient vs. the warmed temperature treatment. We tested for relationships between biomass and the intensity of parasitism by regressing host (or hemiparasite) aboveground (or belowground) biomass against the number of haustorial connections between parasite and host. Finally, to determine how the temperature treatment affected the number of haustoria per hemiparasite, we used a generalized linear model (GLM) with a Poisson distribution.

All analyses were conducted in R version 3.6.0 [27].

## Results

The warmed greenhouse bay achieved significantly higher temperatures than the ambient bay overall during the course of the experiment (16.5 ± 0.1 C vs. 13.9 ± 0.1 C [mean ± SE]; S1 Table). Similarly, both daytime and nighttime temperatures were significantly elevated in the warmed vs. ambient bay (day: 20.5 ± 0.11 C vs. 16.9 ± 0.1 C; night: 13.5 ± 0.1 C vs. 11.7 ± 0.1 C; S1 Table), as were daily maximum and minimum temperatures (maximum: 25.9 ± 0.4 C vs. 20.1 ± 0.1 C; minimum: 10.3 ± 0.2 C vs. 8.7 ± 0.2 C; S1 Table). Although our ambient treatment averaged about 1 C warmer than recent mean July temperatures near the site of *C*. *sulphurea* seed collection (13.9 C vs. 12.7 C), plants in the warmed treatment experienced warming of about 3.8 C on average, close to our target of 3 C.

**Fig 1:**
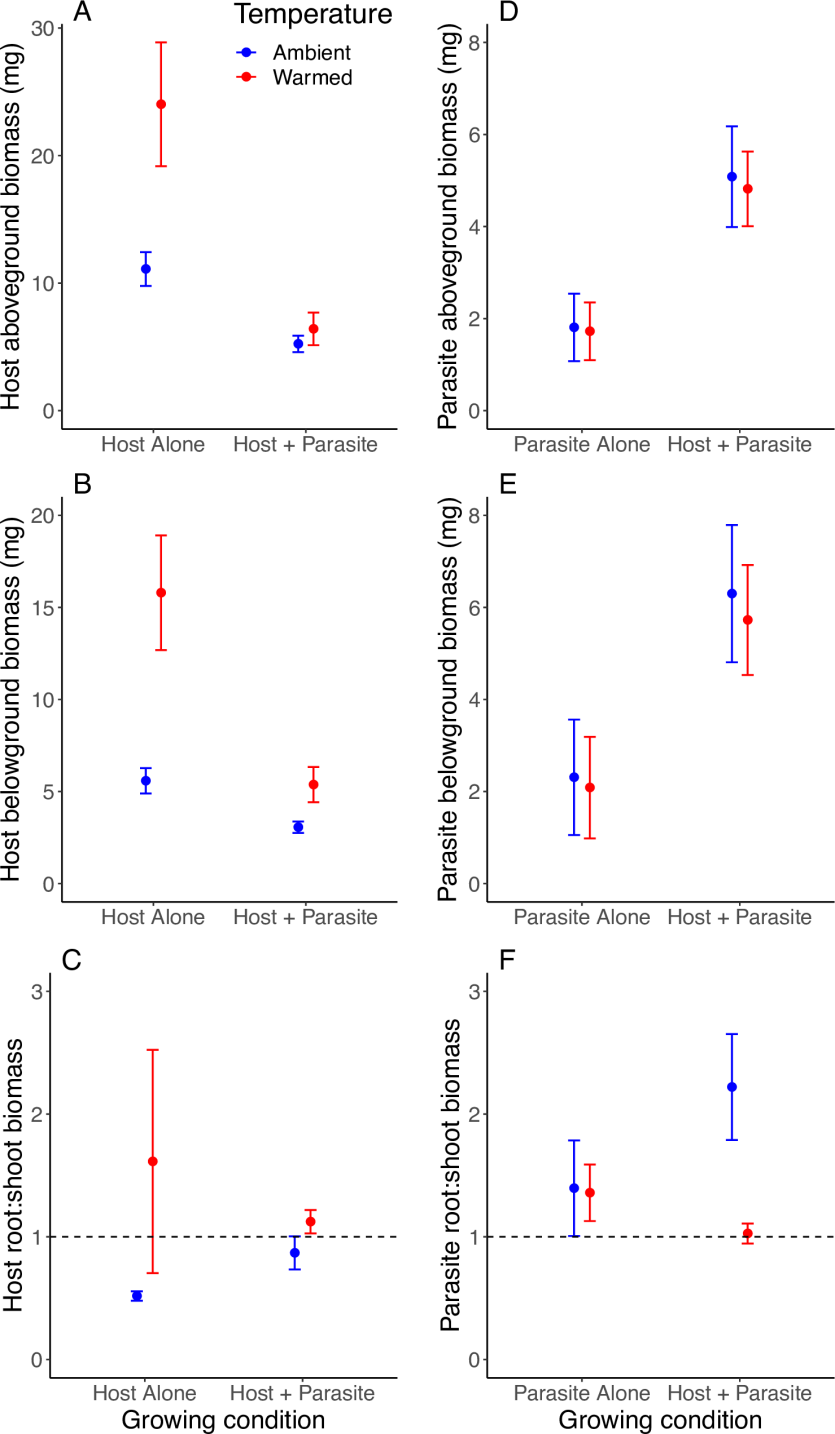
Effects of growing condition (host or parasite alone vs. host + parasite) and temperature treatment (ambient vs. warmed) on (A) host aboveground biomass, (B) host belowground biomass, (C) the ratio of host root to shoot biomass, (D) parasite aboveground biomass, (E) parasite belowground biomass, and (F) the ratio of parasite root to shoot biomass. All values are means ± SE. The dashed lines in (C) and (F) indicate a ratio of 1, equal root and shoot biomass.

Host plants produced more aboveground biomass when grown alone than with a hemiparasite (Table 1), yielding on average three times more biomass (Fig 1A). There was no effect of temperature on host aboveground biomass. Both growing condition and temperature affected belowground biomass (Table 1), with host grasses producing the most biomass when grown alone under warming and the least biomass when grown with a hemiparasite under ambient temperatures (Fig 1B). Mean belowground biomass of grasses grown alone under ambient temperatures did not differ from grasses grown with a hemiparasite under warming (5.58 ± 0.69 mg vs. 5.37 ± 0.96 mg; t_95_ = -0.21, P > 0.84), suggesting that the benefit of warming was negated by the hemiparasite (Fig 1B). Host grass root:shoot ratio increased under elevated temperature and when grown with a hemiparasite compared to when grown alone (Table 1 and Fig 1C). For host grasses grown under ambient temperatures, association with the hemiparasite increased the root:shoot ratio to a more equal allocation between roots and shoots, whereas hosts grown alone invested more in shoot growth (root:shoot = 0.9 ± 0.1 vs. 0.5 ± 0.04; t_95_ = 2.88, P < 0.0049; Fig 1C).

**Table 1:** Best-fitting linear models for aboveground biomass, belowground biomass, and the ratio of belowground to aboveground (root:shoot) biomass of the host and hemiparasite.

Plant	Biomass	Predictor	Estimate^a^	SE	t	df	P
Host (*Bouteloua gracilis*)	Above	GC	-1.03	0.213	-4.84	180	**<0.00001**
	Below	GC	-0.736	0.197	-3.74	179	**0.000252**
		T	0.641	0.186	3.45		**0.000691**
	Root:Shoot	GC	0.314	0.0993	3.16	179	**0.00183**
		T	0.426	0.0936	4.55		**<0.00001**
Hemiparasite (*Castilleja sulphurea*)	Above	GC	0.952	0.320	2.98	155	**0.00334**
		T	0.521	0.271	1.93		0.0560
	Below	GC	1.12	0.310	3.61	156	**0.000417**
	Root:Shoot	GC	0.557	0.211	2.64	154	**0.00920**
		T	-0.511	0.146	-3.51		**0.000588**
		GC×T	0.799	0.301	2.66		**0.00876**

GC, growing condition; T, temperature.

^a^Coefficients for above- and belowground biomass are on a log scale; coefficients for root:shoot biomass are on a logit scale.

Sulphur paintbrush grew more aboveground and belowground biomass when with a host plant vs. alone (Table 1), nearly doubling the total biomass when associated with a host grass (Fig 1D and 1E). For paintbrush with observed haustoria (as described below), plants produced about ten times more biomass on average compared to paintbrush lacking haustoria. We also found a marginally significant positive effect of warming on hemiparasite aboveground biomass (Table 1), with the model including both temperature treatment and growing condition fitting marginally better than the model with only growing condition (LR test: *χ*^2^_1_ = 3.73, P < 0.053). Paintbrush root:shoot biomass increased when plants grew alongside a host, but the effect of temperature on root:shoot biomass depended on growing condition (Table 1). When hemiparasites grew alone, warming did not alter root:shoot biomass, but when grown with a host, warming decreased the root:shoot ratio (2.2 ± 0.4 vs. 1.0 ± 0.1; t_119_ = 3.69, P < 0.00034; Fig 1F).

Within individual plants, aboveground biomass positively correlated with belowground biomass for both hosts (r = 0.88, t_120_ = 20.5, P < 0.00001) and parasites (r = 0.90, t_119_ = 22.0, P < 0.00001). Comparing across the species, host belowground biomass also positively correlated with hemiparasite aboveground biomass (r = 0.21, t_110_ = 2.26, P < 0.026).

**Fig 2:**
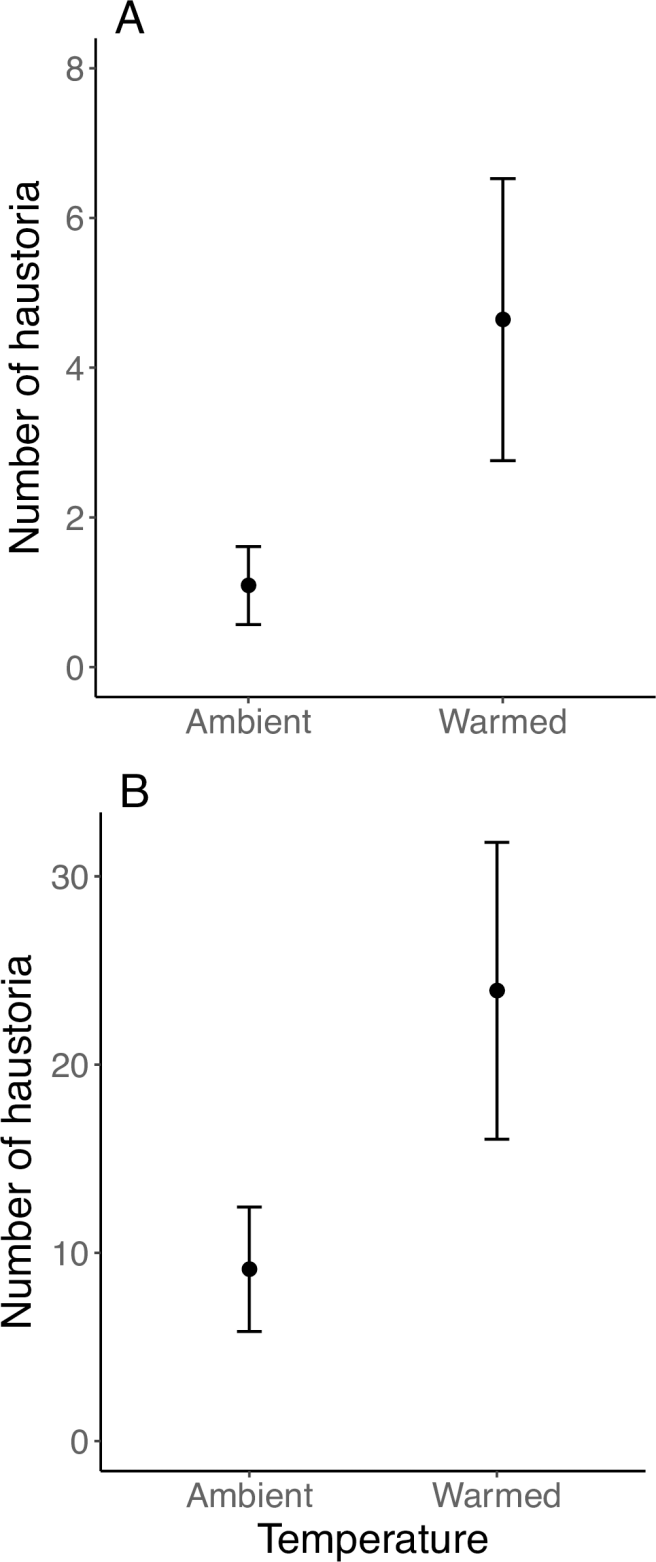
Mean number of haustoria (± SE) by temperature treatment for (A) all hemiparasite plants grown with a host plant (ambient: n = 59, warmed: n = 54) and (B) only those hemiparasite plants that produced at least one haustorium when grown with a host plant (ambient: n = 8, warmed: n = 13).

Altogether, haustorial connections were found on 21 hemiparasites (19%), 13 of which were grown under elevated temperatures. The number of hemiparasites with vs. without haustoria did not differ by temperature treatment (Yates *χ*^2^_1_ = 0.90, P < 0.34). The number of haustorial connections between host and hemiparasite positively correlated with hemiparasite biomass, both aboveground (r = 0.35, t_119_ = 4.12, P < 0.00001) and belowground (r = 0.41, t_119_ = 4.88, P < 0.00001). We also found a positive effect of warming on the number of haustoria, both when considering all hemiparasites (GLM: 1.45 ± 0.13 [estimated model coefficient, mean ± SE], z_132_ = 11.14, P < 0.00001; Fig 2A) and only those that developed at least one haustorium (GLM: 0.96 ± 0.13, z_20_ = 7.41, P < 0.00001; Fig 2B).

## Discussion

To forecast the effects of climate change on these host and parasite species, it is necessary to understand how elevated temperatures affect their interaction. Warming had direct, positive effects on host growth, but the magnitude of the effect depended on interactions with the hemiparasite. Similarly, warming induced the formation of more haustorial connections and affected hemiparasite biomass allocation to roots vs. shoots, but this allocation effect disappeared when hemiparasites were grown alone. To our knowledge, this is the first experimental investigation of how warming affects biomass production of a hemiparasite and its host. Our results reinforce the idea that the same change in climatic conditions can affect interacting species differently, and further indicate that interactions themselves, perhaps especially those that involve physiological linkages as seen in many host-parasite interactions, can modify the responses of species. Therefore, to predict how species will be affected by environmental change, it is important to understand not only the direct effects of warming on species but also the indirect effects that are mediated by species interactions [5].

Rising temperatures generally stimulate carbon gain through photosynthesis until an optimum, thereby increasing plant physiological performance. We observed increased belowground growth and greater allocation of resources to roots in host grasses under elevated temperatures and well-watered conditions (Fig 1B and 1C), in line with evidence that rising temperatures enhance respiration, induce nutrient uptake, and/or increase branching in root architecture to access more resources [28]. However, the increase in belowground biomass was nullified when grasses grew alongside a paintbrush, with host grasses producing almost exactly the same amount of belowground biomass on average when grown alone under ambient conditions vs. together with a parasite under warming (Fig 1B). Reduced biomass production in hosts grown with hemiparasites is expected [29], as *C*. *linariifolia* gains about 40% of its carbon from hosts [30], and root hemiparasites in general construct 20–80% of their biomass from host resources [12]. Grass hosts have also been found to invest more in roots when interacting with *C*. *miniata* [29]. Given that haustoria were found on only 19% of the hemiparasites, it is possible that some of the negative effects on host growth did not result from direct transfer of resources from host to parasite. For instance, parasitic plants in the Orobanchaceae can suppress host photosynthesis [11]. The strongly diminished effect of warming on hosts grown with a hemiparasite may also reflect a greater intensity of parasitism, as hemiparasites produced four times as many haustoria under warming (Fig 2).

Hemiparasites typically show elevated transpiration rates relative to photosynthesis because transpiration drives nutrient uptake (e.g., [31]), leading to the predictions that warming temperatures induce water stress that feeds back to reduce performance or, alternatively, enhanced transpiration increases nutrient uptake [8]. Similar to other *Castilleja* species [29], we found growing alongside a host increased biomass under ambient conditions (Fig 1C). Interestingly, we found warming affected neither the absolute amounts of biomass produced by paintbrush (although there was a marginally significant positive effect of warming on aboveground growth) nor biomass allocation to roots vs. shoots for plants without grass hosts (Fig 1D and 1F). However, for paintbrush grown with a grass, warming reduced investment in root biomass. Thus, paintbrush appears insensitive to the direct effects of warming when grown alone but through indirect signals reduced root investment relative to shoots when a host was present. This may represent a means to take advantage of both the direct stimulation of temperature on growth and the indirect benefit from host resources. But because there was no stimulation in paintbrush grown alone, resource limitations may play a key role in paintbrush response to rising temperatures in the future.

While *Castilleja* species allocate less biomass to roots when grown with a host vs. alone [29], how haustoria determine this pattern is less understood because limited data exist on the number of haustoria relative to performance. We found no haustoria on paintbrush grown alone and greater numbers of haustorial connections under warming, suggesting warming may exacerbate hemiparasite virulence. While we found no effect of warming on belowground biomass, the number of haustoria positively related to both above and belowground biomass, linking parasite performance with greater host connectivity. Whether increased biomass resulted from or enabled greater connectivity is difficult to parse, especially given not all haustoria may be functional [32,33]. Given the duration of the experiment was also short relative to the life cycles of these perennial species, it is possible a stronger relationship between density of haustoria and parasite biomass would emerge over time. Additional study of the relationship between haustoria number and hemiparasite performance, especially under changing resource conditions, may help resolve their contribution to growth.

An advantage of conducting studies on hemiparasite-host interactions in the greenhouse is that we could control whether the species could interact. In the field, it is often difficult to determine whether hemiparasites are connected to hosts or are free-living [15]. Even so, it is possible that we failed to detect haustoria on some plants, especially as haustoria can vary in morphology and size, with small haustoria prone to breakage [34]. Controlled conditions also enabled us to standardize host and parasite age, such that within each group, all plants were at the same developmental stages when assigned to growing condition and temperature treatment. In natural communities, it is possible that host plant and hemiparasite ontogeny and/or phenology could shift such that species become less synchronized as climatic cues change [35]. For example, if the timing of hemiparasite germination shifts earlier but host germination does not, the frequency of interactions may decline, causing the hemiparasite to experience reduced fitness, at least during early growth stages. The idea that climate change can generate phenological mismatches between hosts and parasites is well-established (e.g., [36]) but has not been discussed in the context of host plants and their hemiparasites.

Our findings reveal both direct and indirect effects of warming for this pairwise host-parasite interaction even under simplified environmental conditions. Additional interactions with other community members, including mutualists and competitors, as well as other facets of climate change, such as elevated carbon dioxide and drought, would likely add to the complexity of outcomes [8]. Future studies that measure additional ecophysiological traits, such as water use efficiency, heterotrophy, and autotrophic carbon gain, would be useful for understanding the mechanisms underlying the responses of host and parasite. Such mechanistic knowledge should improve our ability to forecast the effects of climate change on species and their interactions.

## Supporting information

S1 Data Raw data. Data analyzed in this manuscript.
(XLSX)

S1 Fig Haustoria images. Photographs of haustoria (A-D) produced by *Castilleja sulphurea*, taken under a dissecting microscope at 20X magnification. Black arrows point to haustorial connections between *C*. *sulphurea* and *Bouteloua gracilis* roots.
(PDF)

S1 Table Greenhouse temperature treatments. Linear models for greenhouse temperature data, with all data (all), only daytime data (day), only nighttime data (night), daily maximum (maximum), or daily minimum (minimum) as the response variable and temperature treatment (ambient vs. warmed) as the predictor.
(PDF)
