# Supplementary material for: Parasitism modifies the direct effects of warming on a hemiparasite and its host
Source: PLoS One. 2019 Oct 30;14(10):e0224482. doi: 10.1371/journal.pone.0224482 (PMC6821401; doi:10.1371/journal.pone.0224482)

Fig S1. Photographs of haustoria (A-D) produced by *Castilleja sulphurea*, taken under a dissecting microscope at 20X magnification. Black arrows point to haustorial connections between *C. sulphurea* and *Bouteloua gracilis* roots.

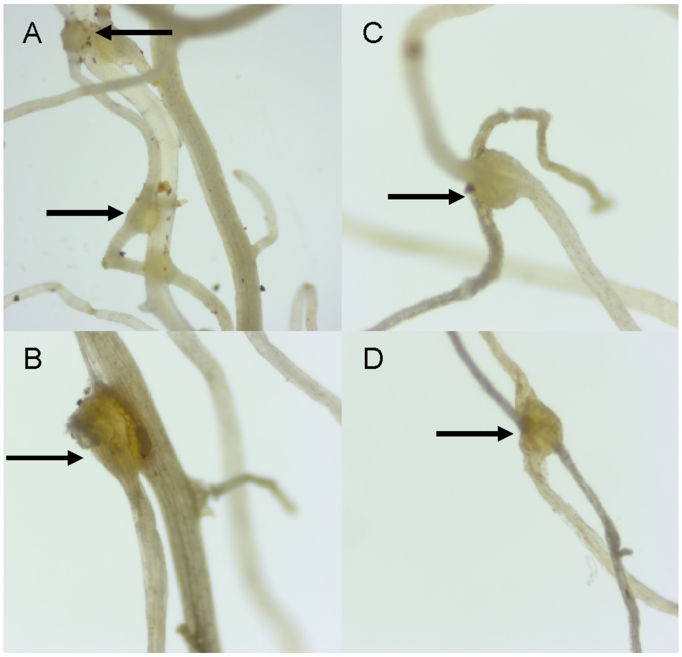

Supplement: S1 Fig — Photographs of haustoria (A-D) produced by Castilleja sulphurea, taken under a dissecting microscope at 20X magnification. Black arrows point to haustorial connections between C. sulphurea and Bouteloua gracilis roots. (PDF) [file pone.0224482.s002.pdf]
