# Supplementary material for: Parasitism modifies the direct effects of warming on a hemiparasite and its host
Source: PLoS One. 2019 Oct 30;14(10):e0224482. doi: 10.1371/journal.pone.0224482 (PMC6821401; doi:10.1371/journal.pone.0224482)
Supplement: S1 Table — Linear models for greenhouse temperature data, with all data (all), only daytime data (day), only nighttime data (night), daily maximum (maximum), or daily minimum (minimum) as the response variable and temperature treatment (ambient vs. warmed) as the predictor. (PDF) [file pone.0224482.s003.pdf]

**Table S1.** Linear models for greenhouse temperature data, with all data (all), only daytime data (day), only nighttime data (night), daily maximum (maximum), or daily minimum (minimum) as the response variable and temperature treatment (ambient vs. warmed) as the predictor.

| Temperature | Estimate | SE | t | df | P |
|---|---|---|---|---|---|
| All | 2.60 | 0.0804 | 32.3 | 13,258 | **<0.00001** |
| Day | 3.61 | 0.104 | 34.7 | 5,668 | **<0.00001** |
| Night | 1.84 | 0.0720 | 25.5 | 7,588 | **<0.00001** |
| Maximum | 5.77 | 0.399 | 14.5 | 138 | **<0.00001** |
| Minimum | 1.53 | 0.326 | 4.69 | 138 | **<0.00001** |
